# Persistent postural‐perceptual dizziness versus vestibular migraine: A narrative review

**DOI:** 10.1111/head.15078

**Published:** 2025-10-28

**Authors:** David Moreno‐Ajona

**Affiliations:** ^1^ Headache Group, NIHR King's Clinical Research Facility & SLaM Biomedical Research Centre King's College London London UK; ^2^ Wolfson SPaRC, Institute of Psychiatry, Psychology and Neuroscience King's College London London UK; ^3^ Joint Neuro‐Otology Clinic, Queen Elizabeth Hospital Lewisham & Greenwich Trust London UK

**Keywords:** functional dizziness, PPPD, vestibular migraine

## Abstract

**Objective:**

This article reviews the differences and similarities between persistent postural‐perceptual dizziness (PPPD) and vestibular migraine.

**Background:**

PPPD is considered a chronic functional vestibular disorder characterized by persistent dizziness, unsteadiness, nonspinning vertigo, and often exacerbated by upright posture, movement, or complex visual stimuli. Frequently misdiagnosed, PPPD shares overlapping features with vestibular migraine (VM), a common cause of episodic vertigo.

**Methods:**

A literature search was conducted covering articles published from January 2000 to March 2025 with a special focus on recent publications that discuss both PPPD and VM.

**Results:**

Although some propose that PPPD is a chronic form of VM, its historical classification as a functional disorder, distinct from the neurological basis of migraine, may suggest otherwise. This review explores PPPD's diagnostic criteria, pathophysiology, and the differential diagnosis of VM, emphasizing the importance of identifying migraine symptoms to guide treatment. Evidence supports migraine prevention and specifically flunarizine as a primary preventive treatment for VM, with emerging data on calcitonin gene‐related peptide‐targeted treatments showing promise. For PPPD, however, vestibular rehabilitation therapy is a cornerstone treatment.

**Conclusion:**

A multidisciplinary approach addressing both conditions is critical for optimal patient outcomes. Studies on comorbid migraine treatment in patients with PPPD are warranted and may reveal distinct phenotypes within the same disease spectrum.

## Plain Language Summary

Dizziness due to persistent postural‐perceptual dizziness (PPPD) and vestibular migraine (VM) are often confused due to similarities in symptoms, making it hard to diagnose and treat these conditions correctly. In this article, we reviewed studies from 2000 to 2025 to compare how these conditions differ in terms of underlying causes, symptoms, and treatments. Results indicated that to date, treatment of PPPD requires balance exercises, whereas VM responds to migraine medicines; a multidisciplinary, tailored approach is essential for managing patients with PPPD and VM, ensuring optimal symptom control and quality of life.

**Abbreviations**
PPPD: persistent postural‐perceptual dizziness
VM: vestibular migraine

## INTRODUCTION

“Psychogenic vertigo” was coined as a term in 1958 and described as a distinct type of vertigo. The authors, an otologist and a psychiatrist, discussed that it is common for vertigo to provoke anxiety but in the case of psychogenic vertigo, this would be contrariwise. They included 34 patients in a study and discussed that external or spinning vertigo would be more consistent with an organic cause, in comparison to nonspinning, internal vertigo, more typical of psychogenic vertigo.^1^ They also discussed a hysterical or phobic nature of the symptoms, mentioning the classical *la belle indifférence* ^2^ observed in some of the patients. The use of some of these terms and overly simplistic association with anxiety would have likely led to stigmatization. Over time, other terms such as psychosomatic or somatoform dizziness were used in the literature to refer to the same condition, whereas the more contemporaneous “functional dizziness or vertigo” also encompass phobic postural vertigo,^3^ chronic subjective dizziness,^4^ and space‐motion discomfort.^5^ Similarly, in very visually dependent patients with a history of a peripheral vestibular disorder, “visual vertigo,” or dizziness provoked by moving objects or patterns have been described.^6^ In an effort to unify diagnostic criteria and describe a very frequent phenotype encountered in neuro‐otology consultations, persistent postural‐perceptual dizziness (PPPD) was defined by the Bárány Society in 2017.^7^ Notably, vertigo generally may not be a rotatory sensation. Indeed, the Bárány society defines external vertigo as well as spontaneous internal spinning or nonspinning vertigo, the latter being a rocking or swaying sensation.^8^

PPPD is described by the International Classification of Vestibular Disorders as chronic vestibular disorder characterized by persistent sensations of dizziness, unsteadiness, or nonspinning vertigo lasting 3 months or more.^7^ Symptoms are typically exacerbated by upright posture, active or passive motion, and exposure to complex visual environments (visual vertigo). It is reported that PPPD may follow an acute vestibular event, such as vestibular neuritis, benign paroxysmal positional vertigo, or even vestibular migraine (VM), but may also arise from psychological stressors or without a clear trigger.^7^, ^9^

VM is considered the leading cause of episodic vertigo and is characterized by vertigo episodes associated with migrainous features such as migrainous headache, photophobia, or phonophobia.^10^ The diagnostic criteria for VM have been harmonized between the Bárány Society and the appendix of the ICHD‐3 more recently,^11^ ensuring consistency in identifying definite VM based on episode duration, migraine history, and associated symptoms. Both internal and external vertigo can be present in VM.^11^

Although VM is considered the most common cause of episodic vertigo,^10^ PPPD is considered the most common cause of persistent dizziness.^12^ Indeed, some researchers hypothesize that PPPD may represent a chronic manifestation of VM, given their frequent coexistence and similar phenotype.^13^ However, PPPD's roots in functional dizziness—implying a maladaptive response rather the disorder being caused by a structural lesion^14^—may contrast with VM's basis within the broader migraine pathophysiology.^15^

This review aims to clarify the differential diagnosis of PPPD and VM, and explore their relationship, phenotypic overlaps, as well as responses to treatment.

## METHODS

### Search strategy

To conduct this narrative review, a literature search was conducted using PubMed and Web of Science databases, covering articles published from January 2000 to March 2025. Search terms included keywords “persistent postural‐perceptual dizziness,” “functional dizziness,” “vestibular migraine,” and “migraine associated dizziness.”

### Selection criteria

Included articles in this review were peer‐reviewed studies, reviews, and clinical trials in English focusing on PPPD and VM pathophysiology, diagnosis, and treatment. Publications from the last 10 years, commonly referenced and most relevant to this current review if they discussed both PPPD and VM, were prioritized. Articles not included in this narrative review included non‐English articles and case series with fewer than five patients.

Data from the eligible articles were collected, and information was collated to summarize epidemiology, diagnostic criteria, pathophysiology, differential diagnosis, and treatment outcomes.

## RESULTS

Following search and selection, 58 scientific articles on PPPD and VM were included (Table 1).

**TABLE 1:** Diagnostic criteria.

PPPD The ICVD outlines five diagnostic criteria for PPPD, all of which must be met^7^:	VM The Bárány Society and appendix of ICHD‐3 criteria for definite VM require^11^, ^16^:
Dizziness, unsteadiness, or nonspinning vertigo^a^ present on most days for ≥3 months. Symptoms exacerbated by upright posture, active/passive motion, or complex visual stimuli. Triggered by a vestibular, neurological, or psychological event. Symptoms cause significant distress or functional impairment. Symptoms not better explained by another disorder.	At least five episodes of vestibular symptoms^b^ lasting 5 min to 72 h. Current or past history of migraine with or without aura. One or more migraine features (migrainous headache, photophobia, phonophobia, or visual aura) with at least 50% of episodes Symptoms not better accounted for by another diagnosis.

Abbreviations: ICVD, International Classification of Vestibular Disorders; PPPD, persistent postural‐perceptual dizziness; VM, vestibular migraine.

^a^ PPPD symptoms described in the ICVD include nonspinning vertigo, dizziness (impaired spatial orientation), unsteadiness, distorted sensations of swaying, rocking, bobbing, or bouncing of oneself.^7^

^b^ Vestibular symptoms in VM include spinning vertigo, nonspinning vertigo, postural vertigo as well as visual vertigo.^11^, ^16^

### Epidemiology

PPPD is a common diagnosis encountered in specialized dizziness clinics, accounting for approximately 10% of cases as a primary diagnosis, and up to 20% when coexisting with other vestibular disorders. It is considered the first cause of persistent dizziness.^12^ It predominantly affects women (2:1 ratio), and has a peak onset between 30 and 50 years.^17^ VM affects approximately 1% of the general population, with a higher prevalence in women (3:1) and a typical onset in the third to fourth decade.^18^ Up to 55% of patients with migraine report vestibular symptoms,^10^ and VM is considered a frequent trigger for PPPD, implicated in 15%–20% of cases.^13^

### Pathophysiology

#### PPPD

PPPD is considered a functional vestibular disorder arising from maladaptive changes^19^, ^20^ in postural control and multisensory integration.^21^ Neuroimaging suggests altered connectivity in visuo‐vestibular and anxiety networks, with heightened sensitivity to motion and visual stimuli.^22^ Despite various studies in line with this hypothesis, linking visual and emotional processing,^23^ and an overreliance on visual information for spatial orientation (visual dependence),^24^, ^25^ the question on whether the anxiety and hyper alert state are causative or a consequence of the symptoms has not been fully answered. Personality traits like neuroticism and introversion are thought to predispose individuals to PPPD, perpetuating a cycle of hypervigilance and symptom amplification.^26^ A structural neuroimaging study used cortical surface‐based morphometry to compare patients with PPPD against healthy nondizzy controls who were matched by personality traits, anxiety, and depressive symptoms. This showed abnormal cortical folding in vestibular and somatosensory spatial association cortices.^27^ A recent functional magnetic resonance imaging study showed altered network connections between the visual cortex and sensorimotor and multisensory vestibular cortices with a change in the topological properties, with lower efficiency in PPPD than healthy controls following graph theoretical analysis. This theoretical analysis models the brain as a network measuring distance between brain regions and how synchronized is their activity as per correlations in functional magnetic resonance imaging study signal, so that more efficient effectively means shorter path lengths between brain regions.^28^

An excellent review on the current understanding of PPPD pathophysiology was conducted by Lee et al.^12^ PPPD was conceptualized again as a functional neuro‐otologic disorder resulting from maladaptive responses to triggering events, such as vestibular, medical, or psychological insults.^29^ Three generations of pathophysiological models are discussed.^12^ The first generation, an anxiety‐driven postural control, focuses on anxiety as a causative factor heightening sensitivity to motion stimuli and over‐reliance on visual or somato‐sensory inputs for balance, which would perpetuate symptoms.^30^ Early studies on phobic postural vertigo linked this to an anxiety‐driven balance disturbance,^31^ in line with what was discussed in 1958.^1^ The second generation, based on predictive processing and sensory mismatch, relied on the brain's predictive coding failure to adapt post‐trigger exposure, leading to persistent misperceptions of motion or unsteadiness. Patients would show reduced habituation to visual motion and increased visual dependence.^32^, ^33^, ^34^, ^35^ In line with this model, a Bayesian predictive processing model suggests excessively precise prior beliefs about body motion or threat, influenced by traits like neuroticism, drive perceptual errors, reinforcing dizziness.^12^, ^36^ The third‐generation model prioritizes postural stability integrating physiological, neuroimaging, and psychological data.^12^ Following a trigger, the brain would adopt a high‐stakes postural control strategy prioritizing rigid stability to prevent falls, leading to over‐reliance on visual inputs and reduced vestibular integration.^37^, ^38^ Multisensory processing alterations potentially due to reduced cortical folding and gray matter volume^27^, cortical network shifts with increased connectivity between the prefrontal cortex (regulating attention and emotion), and primary visual/motor regions would perpetuate symptoms.^39^

The third‐generation model supports the current multimodal treatment approach for PPPD, based on vestibular rehabilitation, cognitive behavioral therapy, and, in some cases, serotonergic medications.^21^

#### VM

Our current understanding is that VM pathophysiology is, reasonably, that of migraine generally, involving cortical hyperexcitability and dysregulation of the trigemino‐vascular system, with calcitonin gene‐related peptide (CGRP) playing a central role.^15^, ^40^, ^41^ In rodent models, the vestibular nucleus has shown to express CGRP.^42^ Recently, the inhibition of direct glutamatergic connections between the trigeminal nucleus caudalis (known to be linked to migraine pathophysiology)^15^ and the vestibular nucleus led to alleviation of vestibular dysfunction in rats chronically exposed to nytroglycerin.^43^ At the cortical level, we know the tempo‐parietal junction is key for sensory integration including visuo–spatial functions. There is considerable connection between the vestibular system, thalamus, and temporoparietal cortex.^44^ Despite vestibular symptoms not being considered canonical in migraine (unlike photophobia, phonophobia, movement sensitivity, or nausea), up to half of patients with migraine might report vestibular symptoms when asked.^10^ The most plausible hypothesis suggests that vestibular symptoms in migraine likely stem from the activation of vestibular nuclei and thalamocortical pathways during migraine attacks.^44^ VM as a distinct diagnosis differentiated from migraine generally, may have a practical utility when considering clinical trial design, and most bothersome symptoms. It is worth noting that the current diagnostic criteria for VM do not specify that the vestibular symptoms are the most bothersome symptoms (ICHD‐3).^11^

From a diagnostic perspective, unlike PPPD, VM is considered an episodic disorder, and if VM symptoms become chronic or constant, the diagnosis of PPPD instead of chronic VM has been proposed as being more suitable.^13^ It is also argued that VM can potentially be another trigger for PPPD or pose a frequent comorbidity.^45^ It is therefore unclear if these are two manifestations of the same disease spectrum, if they are commonly comorbid, or they are distinct pathophysiological entities that should be managed with separate approaches.

### Pathophysiological differences and similarities

Theoretically, the pathophysiology of PPPD could share some similarities with VM, given shared symptoms like dizziness and visual sensitivity. However, different key mechanisms and underlying drivers have been proposed for both entities.^29^, ^44^ Both disorders involve altered visuospatial perception and multisensory integration,^23^, ^46^ with patients with PPPD showing heightened visual dependence and reduced vestibular cortical connectivity,^47^ and patients with VM exhibiting impaired visuospatial processing due to dysfunctional vestibular–visual interactions during migraine attacks.^48^ In VM, cognitive symptoms have been described,^49^, ^50^ and it is typical for patients with migraine generally to describe “brainfog.”^51^ PPPD has also been linked to subjective cognitive dysfunction that was predicted by duration of symptoms and intensity of dizziness, measured with the dizziness handicap inventory (DHI).^52^ Unlike PPPD, VM may be driven by a hyperexcitable cerebral cortex, with increased activity in visual and vestibular cortical areas (e.g., temporoparietal junction)^53^ and altered thalamo‐cortical networks, leading to sensory amplification and episodes of vestibular symptoms, with or without headache.^54^ In contrast, PPPD is described as a functional, maladaptive response to a trigger, with decreased vestibular cortical activity and a persistent shift toward rigid postural control perpetuated by anxiety.^55^ Although the pathophysiology of VM may be tied to neurochemical factors such as serotonin,^56^ dopamine,^57^ CGRP,^40^ pituitary adenylate cyclase‐activating polypeptide,^58^ vasoactive intestinal peptide,^59^ nitric oxide,^60^ and even amylin dysregulation,^61^ as well as genetic factors,^62^ PPPD's proposed mechanisms are more closely associated with learned behavioral adaptations^63^ and psychological traits like neuroticism.^26^ Thus, whereas VM may reflect a disorder of organic cortical hyperexcitability,^64^ PPPD is considered a chronic, functional, maladaptation disorder.^63^ Given these clear differences, it is theoretically possible for patients to experience both conditions and for them to be comorbid.

To the extent that this is possible in clinical practice, however, we frequently try to identify one cause that can explain all patients' symptoms,^65^ so we can adopt suitable treatment approaches. Although VM is defined as an episodic disorder, the pathophysiological difference between episodic migraine, the so called high‐frequency episodic migraine, and chronic migraine may be insignificant. It seems plausible that that those with 8 headache days a month, 14 headache days a month, and more than 15 headache days a month are biologically similar. However, contrary to this, we know that people who experience high‐frequency episodic migraine are at higher risk of developing chronic migraine,^66^ and migraine frequency can naturally vary month to month so that some patients fluctuate from the episodic to chronic state, thus the classification of VM as a purely episodic disorder is likely simplistic. Migraine chronification has been linked to medication overuse^67^ as well as to allodynia,^68^ a marker of central sensitization.^69^ A recent study by Abdulla et al.^70^ demonstrated that cranial allodynia is significantly increased in patients with VM compared to patients with migraine without vestibular symptoms and other vestibular conditions, with 68% of patients with VM reporting allodynia during attacks. Patients with chronic migraine commonly present with comorbid increased anxiety and depression,^71^ even psychological traits like neuroticism, although these are typically not described as triggering factors in migraine generally.^72^ People who experience chronic migraine can indeed develop a phenotype with daily and even persistent symptoms.^73^ New daily persistent headache (NDPH)^11^ has also been discussed as an entity with different phenotypes, one of them with more migraine‐related symptomatology. The latter would respond better to anti‐migraine therapies, compared to the more featureless form.^74^ Other noncanonical symptoms in migraine, cognitive impairment, or brain‐fog, which we understand as a premonitory‐like symptom,^15^ can also be described by people who experience migraine as a constant symptom at any phase of the attack or even in the absence of headache interictally.^75^ Therefore, it seems likely that a chronic or persistent form of VM could form a feasible entity. Additionally, in line with what we define as NPDH, phenotypically, some patients report new daily persistent dizziness that may be a particular entity that deserves further investigation.^76^

### Differential diagnosis

As discussed above, the overlap in dizziness, visual sensitivity, and unsteadiness complicates distinguishing PPPD from VM. Current differentiators may include:

- Symptom pattern: PPPD presents with persistent, daily symptoms,^7^ whereas VM is typically episodic, with symptom‐free intervals.^77^ Chronic VM, however, may blur this distinction if identified as a separate entity.^13^
- Migrainous features: VM is associated with migrainous headache, photophobia, phonophobia, or visual aura. Nevertheless, up to 53% of patients with PPPD may have coexisting migraine.^78^
- Triggers: PPPD symptoms are consistently provoked by upright posture, motion, or visual stimuli,^7^ whereas VM episodes may occur spontaneously or with migraine triggers (e.g., menstruation and relaxation after stress).^79^ Nonetheless, premonitory symptoms may be confounded with perceived migraine triggers.^80^

Other differential diagnoses for PPPD include benign paroxysmal positional vertigo, Meniere's disease, vestibular neuritis, and bilateral vestibulopathy.^81^ A positive diagnosis for functional disorders is currently recommended, avoiding reliance on unnecessary tests.^82^ Increased antero–posterior sway in posturography has been identified in PPPD^83^ and, in general, lower thresholds of vestibular motion perception.^84^ On the other hand, a migraine diagnosis is made clinically.^11^ VM should be distinguished from migraine with brainstem aura, far less common, where theoretically cortical spreading depression is taking place in the brainstem,^11^ although a cortical origin has been hypothesized as well.^85^ As per the diagnostic criteria of migraine with brainstem aura, at least two symptoms are required: dysarthria, vertigo, tinnitus, hypoacusis, diplopia, ataxia, or reduced level of consciousness.^11^

If abnormalities are found in the neuro‐otological examination, comprehensive vestibular testing^86^ (e.g., videonystagmography, caloric testing, video‐head impulse test, and visually evoked myogenic potentials) may be helpful. Vestibular tests may be abnormal in patients with VM, with uncompensated vestibular hypofunction in some cases.^87^ Symptom aggravation following caloric testing is frequent in VM.^88^

### Treatment

#### VM

Traditional migraine preventives are used for the preventive treatment of VM although this is mostly based on open‐label studies, with some recent useful guidelines.^89^ Propranolol is discussed as a first‐line treatment in the literature,^89^ given good tolerability and adherence, although the dizziness handicap inventory (DHI) did not change in the largest controlled trial on metoprolol.^90^ Venlafaxine is discussed as a good alternative treatment^91^, ^92^ if the patient has comorbid low mood, although withdrawal syndrome is reported as a complication by some authors following treatment cessation.^89^ Topiramate, valproate, and amitriptyline are also used but lack robust evidence.^89^, ^91^, ^93^ Unlike other migraine preventives, flunarizine is supported by a controlled study as an effective preventive treatment for VM.^94^ A single‐center observational study reported 90% of patients with VM on flunarizine (10 mg daily) experienced symptomatic improvement, compared to 32% on other medications (e.g., propranolol, topiramate).^95^ The most common flunarizine side effects include tiredness, mood changes, and weight gain although only 10.5% of 200 patients with migraine in our tertiary headache clinic stopped the treatment because of these.^96^ Preliminary data suggest that onabotulinumtoxinA as per the PREEMPT protocol^97^ may be an option for patients with VM,^98^ although the effect seems higher in reducing pain and it has been recommended in patients with more severe headaches.^99^

Recently, a CGRP monoclonal antibody, galcanezumab, was evaluated in the INVESTMENT study, a placebo‐controlled trial for VM.^100^ Over 3 months, galcanezumab (240 mg initial dose, then 120 mg monthly subcutaneously) reduced vestibular migraine patient assessment tool and handicap inventory scores by 14.8 points (vs. 5.1 for placebo, *P* = 0.044), DHI scores by 22.0 points (vs. 8.3, *P* = 0.018), and definite dizzy days from 17.9 to 6.6 (vs. 18.0–12.5, *P* = 0.026). No serious adverse events were reported, suggesting CGRP blockade as a promising therapy for VM.^100^

No specific treatment is recommended for the acute treatment of VM in the International Headache Society guidelines.

#### PPPD

Vestibular rehabilitation therapy (VRT) involves exercises to desensitize patients to motion and visual stimuli and is a cornerstone of PPPD management.^101^ A 2014 study demonstrated 60%–80% symptom reduction with 3–6 months of VRT, improving mobility and reducing anxiety in PPPD.^102^ Cognitive behavioral therapy addresses hypervigilance and maladaptive behaviors, with moderate short‐term benefits.^101^ Early intervention may prevent PPPD chronicity.^103^ A combination of rehabilitation and a psychological intervention, “cognitive physical therapy,” may become as more specific treatment strategy for PPPD.^104^ In line with this, the INVEST trial tested a psychologically informed vestibular rehabilitation program, compared to the standard physiotherapy, with promising results.^105^ Regarding pharmacotherapy, selective serotonin reuptake inhibitors and serotonin‐norepinephrine reuptake inhibitors may reduce anxiety and vestibular symptoms, even in patients without psychiatric comorbidities.^101^, ^106^ No specific medications are approved for the treatment of PPPD specifically.

Interestingly, venlafaxine may be useful for both PPPD^106^ and VM,^91^, ^92^ presumably given the utility of the drug in the treatment of anxiety^107^ and its evidence as a migraine preventive.^108^ A multidisciplinary approach integrating neurotology, neurology, and psychology may optimize outcomes for affected patients.

## DISCUSSION

The debate over whether PPPD represents a chronic form of VM stems from their frequent coexistence and symptomatic overlap.^9^ VM is considered a common precipitant of PPPD, and it has been reported that up to 17% of patients with PPPD meet criteria for VM.^4^ Patient‐reported outcomes such as the DHI have been used to score dizziness symptoms in both VM and PPPD.^50^, ^109^ Specifically, the Niigata PPPD Questionnaire was designed as a useful tool to detect the presence of PPPD‐like symptoms.^110^ However, the Niigata PPPD Questionnaire in patients with VM may demonstrate scores comparable to those in patients with PPPD, suggesting shared sensory processing abnormalities, which further complicates differential diagnosis^111^ and may suggest that the two disorders may form part of the same disease spectrum.

In the past, migraine‐anxiety related dizziness was proposed as a new disorder stemming from the convergence of migraine and anxiety to produce chronic dizziness.^112^ It should be noted that this overlap is no longer in vogue, and VM and PPPD are currently still considered distinct entities with different pathophysiological bases, as discussed above.

Recently, a narrative review by Klein and Schankin^113^ proposes that PPPD and visual snow syndrome (VSS) may both arise from migraine‐related developed sensitization, suggesting they lie on a spectrum of perceptual disorders characterized by hypersensitivity to sensory stimuli and both share migraine as a common comorbidity. This hypothesis aligns with the high prevalence of migraine in both conditions, with 53% of patients with PPPD^78^ and up to 50% of patients with VSS reporting migraine headaches.^114^ Migraine's multisensory hypersensitivity, mediated by thalamocortical dysrhythmia and altered salience network connectivity, may feasibly predispose individuals to persistent vestibular (PPPD) or visual (VSS) symptoms.^113^ Although this perspective supports the association between PPPD and VM discussed here, the current understanding of PPPD is that this is as a functional disorder due to its maladaptive sensory integration,^29^ whereas VSS is recognized as having a distinct pathophysiology with widespread disturbance in the functional connectivity of several brain systems^115^ demonstrated on functional neuroimaging studies, and is currently no longer considered a functional neurological disorder. This distinction highlights the need for precise diagnostic criteria and perhaps the inclusion of chronic VM as a distinct entity or the term “PPPD with or without migraine” to avoid confounding different pathophysiologies.^21^, ^69^ More studies in patients with VM who eventually develop a chronic form are warranted to characterize these differences and similarities.

As previously discussed, galcanezumab has shown efficacy in reducing vestibular symptoms in patients with VM.^100^ This finding suggests a role for CGRP in vestibular symptom generation, warranting further research into its application in chronic vestibular patients with migrainous features. Nitroglycerin, a well‐documented migraine trigger, was able to provoke allodynia and vertigo in more than 50% of patients with migraine,^116^ alluding to shared mechanisms between trigeminovascular nociception and vestibular mechanisms.

The interplay between PPPD and VM necessitates a nuanced approach, as vestibular rehabilitation may worsen migraine symptoms if not carefully tailored, given movement sensitivity is a canonical symptom of migraine,^11^ yet is the first‐line treatment for PPPD. It would be relevant to determine whether preventive treatment of VM, even when not the primary cause, may enhance VRT outcomes in patients with PPPD, particularly in those with other migrainous symptoms.

If patients present with the same phenotype but different pathophysiology, the diagnosis becomes more challenging. However, deep phenotyping and history‐taking (considering not only canonical symptoms but also premonitory‐like symptoms, postdrome, and family history) may reveal the presence of migraine biology or predisposition that would favor migraine treatment in the first place. More studies addressing this question are necessary. It is possible that migraine predicts the development of a PPPD‐like phenotype following a vestibular insult. If VM can be chronic and persistent, some patients may be misdiagnosed with a nonorganic functional neurological disorder leading to psychological harm^117^ and treatment delay. More studies comparing PPPD with and without migraine features would help determine if these are different phenotypes of the same disease spectrum favoring treatment for both VM and PPPD and potentially improving outcomes.

## CONCLUSION

PPPD and VM are vestibular disorders that require careful differential diagnosis. Screening for migraine symptoms is critical, as VM may respond to preventive medications like flunarizine^94^ and potentially CGRP therapies.^100^ PPPD, conversely, benefits from vestibular rehabilitation and psychological input.^101^ Although VM is considered a common cause of episodic vertigo,^10^ and PPPD is considered the most common cause of persistent dizziness,^12^ the symptom overlap seems obvious, and the distinction is not always clear. If PPPD is simply a chronic form of VM, this opens the question on whether this can be correctly called a functional neurological disorder. Conversely, if chronic vestibular migraine was differentiated from PPPD, two different forms of chronic dizziness with similar symptoms would be identified, perhaps adding confusion. Studies on comorbid migraine treatment in patients with PPPD are warranted and may reveal distinct phenotypes within the same disease spectrum. A multidisciplinary, tailored approach is essential for managing patients with PPPD, VM, a chronic form of VM, or both conditions, ensuring optimal symptom control and quality of life.

## AUTHOR CONTRIBUTIONS

**David Moreno‐Ajona:** Conceptualization; methodology; investigation; validation; writing – original draft; writing – review and editing.

## CONFLICT OF INTEREST STATEMENT

**David Moreno‐Ajona** has no conflicts of interest to declare.
